# Full incorporation of Strattice™ Reconstructive Tissue Matrix in a reinforced hiatal hernia repair: a case report

**DOI:** 10.1186/1752-1947-6-234

**Published:** 2012-08-09

**Authors:** Bruce E Freedman

**Affiliations:** 1 Scottsdale Healthcare, 10250 N. 92nd St. Suite 307, Scottsdale, AZ, 85258, USA

## Abstract

**Introduction:**

A non-cross-linked porcine acellular dermal matrix was used to reinforce an esophageal hiatal hernia repair. A second surgery was required 11 months later to repair a slipped Nissen; this allowed for examination of the hiatal hernia repair and showed the graft to be well vascularized and fully incorporated.

**Case presentation:**

A 71-year-old Caucasian woman presented with substernal burning and significant dysphagia. An upper gastrointestinal series revealed a type III complex paraesophageal hiatal hernia. She underwent laparoscopic surgery to repair a hiatal hernia that was reinforced with a xenograft (Strattice™ Reconstructive Tissue Matrix, LifeCell, Branchburg, NJ, USA) along with a Nissen fundoplication. A second surgery was required to repair a slipped Nissen; this allowed for examination of the hiatal repair and graft incorporation 11 months after the initial surgery.

**Conclusion:**

In this case, a porcine acellular dermal matrix was an effective tool to reinforce the crural hiatal hernia repair. The placement of the mesh and method of fixation are believed to be crucial to the success of the graft. It was found to be well vascularized 11 months after the original placement with no signs of erosion, stricture, or infection. Further studies and long-term follow-up are required to support the findings of this case report.

## Introduction

A large hiatal hernia is an acquired diaphragmatic defect with migration of the gastroesophageal junction or variable amounts of stomach with or without intra-abdominal organs. Serious complications such as strangulation, necrosis, or gastric perforation can occur. Surgery is usually recommended for types II, III, and IV hiatal hernias. Hiatal hernia repair is associated with a high rate of recurrence (9 to 44%) [1,2]. The high recurrence rate has led to the use of mesh reinforcement of the crural repair [3,4]. Several types of synthetic (polytetrafluoroethylene, polypropylene, Dacron™) and biological (Strattice,™ Alloderm,™ Surgisis,™ Permacol™) matrices, both allogeneic and xenogeneic, have been applied for the reinforcement of hiatal hernia repair. Synthetic meshes used for hiatal hernia repair are permanent and non-absorbable whereas biological meshes are integrated into the body. Synthetic meshes can elicit a foreign body response that results in encapsulation. Foreign bodies can cause long-term infection and extrusion of the implant. The inflammatory response can result in reabsorption of the implant and deposition of scar tissue, which does not have the native structure, function, or physiology of the original host tissue. The processing of acellular dermal matrices leaves the structural architecture intact. Acellular dermal matrices provide scaffolding for tissue growth with continued reinforcement, and are readily accepted by the body without an immunological or foreign body response [5]. This generates tissue that more closely resembles the lost or damaged tissue.

Although biological meshes are often used in other parts of the abdomen (incisional and ventral hernias), the constant movement due to respiration at the esophageal hiatus has led to concerns over the safety of meshes in these cases [6]. Stadlhuber *et al*. have reported mesh complications in 28 cases after the use of prosthetic reinforcement. No direct cause for erosion, distal esophageal stenosis, and dense adhesion formation could be determined. Both the technical aspects of graft placement and the type of material used were thought to play an important role in the success of the repair [7]. Many studies have shown increased complications with the use of synthetic grafts for hiatal hernia repair [8]; however, there is a paucity of data on the long-term use of biological grafts at the hiatus [9,10]. In this case report I describe the use of a non-cross-linked porcine acellular dermal matrix (PADM) for a large hiatal hernia repair which was evaluated *in situ* 11 months after placement. It is noteworthy that the mesh was shown to be intact and well vascularized with no signs of erosion, stricture, or infection11 months after placement. To the best of my knowledge this is the first case in which revascularization was observed in a patient several months after surgery.

## Case presentation

**Figure 1:**
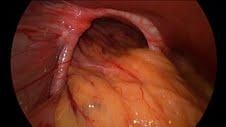
Type III complex paraesophageal hiatal hernia.

A 71-year-old Caucasian woman presented with acid reflux symptoms of substernal burning and significant dysphagia. A barium swallow and endoscopy showed distal esophageal narrowing and a hiatal hernia. A repeat upper gastrointestinal (GI) series revealed a type III complex hiatal hernia with a significant paraesophageal component (Figure 1). The paraesophageal component was causing the distal esophageal narrowing, which was confirmed by a computed tomography scan. Surgery was recommended because her symptoms were caused by both gastroesophageal reflux disease (GERD) and the external compression of the distal esophagus from the paraesophageal component.

**Figure 2:**
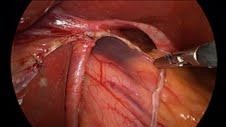
a: Dissecting out the hiatal hernia. b: Hiatus dissected out.

**Figure 3:**
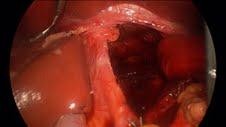
Primary repair of hiatal hernia.

**Figure 4:**
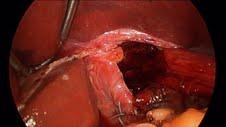
Modified U shape of the porcine accellular dermal matrix graft.

**Figure 5:**
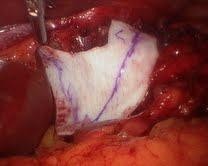
**a: Suturing in Strattice™ at the apex of the crural repair.b**: Top stitch at superior portion of the crural repair.

**Figure 6:**
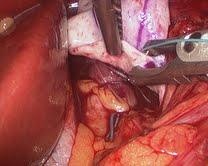
Strattice™ graft cruraplasty.

The patient underwent laparoscopic reduction and repair of the complex type III hiatal hernia with a Nissen fundoplication to treat GERD. She had a large hiatal defect (6cm) with what appeared to be a complex hernia with both paraesophageal and sliding components. The posterior esophageal dissection cleared the hiatus (Figure 2), but the diaphragm and both crura were extremely thinned out. Primary repair was carried out using O Ethibond sutures (ETHICON, Somerville, NJ, USA); however, owing to the condition of the hiatus it was reinforced with an 8 × 8cm piece of Strattice™ xenograft (Figure 3). The graft was cut into a modified U shape with very short arms in the U portion (Figure 4). The O Ethibond suture was used at the apex of the crural repair inferiorly up at the superior portion of the crural repair (Figure 5), and then on the left upper side. The area on the right side was insufficient to suture the overlay patch; therefore ARTISS fibrin glue (Baxter, Deerfield, IL, USA) was used underneath the graft to stick it to the underlying fascia (Figure 6). A posterior stitch was placed in the posterior fundus for marking. The fundus was brought around the retroesophageal space and the fundoplication was carried out. The patient tolerated the procedure well and was transferred to the recovery room in a stable condition.

The patient was discharged on a soft diet and she did well over the ensuing months. Approximately 11 months after the initial surgery she was admitted to my hospital with severe nausea and vomiting. An upper GI series and subsequent endoscopy revealed a slipped Nissen, with the wrap around the proximal stomach and not at the esophagogastric junction.

**Figure 7:**
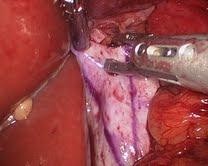
Strattice™ Reconstructive Tissue Matrix 11 months after initial repair.

She underwent laparoscopic revision of the wrap. The previously placed PADM was well incorporated overlying the hiatus and it actually looked like normal fascia. It was still intact and well vascularized with minimal adhesions (Figure 7). The few adhesions were taken down with a suction device. The hiatus did dilate anterior to the graft, which was closed with three interrupted 0 Ethibond sutures to make the hiatus secure. At this point the slipped Nissen was repaired. Postoperative upper GI was normal and the patient has continued to progress over the ensuing nine months.

## Discussion

In cases of hiatal hernias, repetitive stresses at the esophageal hiatus such as coughing, straining, sneezing, and laughing can lead to a very high rate of hernia recurrence. Recurrences of hiatal hernias can be attributed to many factors; however, the absence of a strong fascia at the hiatus is believed to lead to suture pull-out. The application of grafts to reinforce the hiatal repair has resulted in a decrease of hernia recurrence [3]. Several controversies are associated with graft reinforcement: indication for graft placement, the type of graft used, the placement of the graft relative to the hiatus and esophagus, and how the graft is anchored. Synthetic non-absorbable meshes have been shown to cause many complications such as erosion into the stomach or esophagus, adhesions, mesh contraction, fistulas, and strictures [6,7,11]. The severity of these complications has led to the development of alternatives that resist visceral erosions, provide scaffolding for tissue growth, and continue to reinforce the hiatal repair. Biological grafts are ideal for this purpose. Unfortunately, there are few long-term studies on the use of biological grafts at the esophageal hiatus.

A study has compared patients who received a primary hiatal hernia repair versus a primary repair reinforced with a non-cross-linked porcine small intestine submucosa (SIS). They initially reported that the biologic prosthesis reduced hernia recurrence after six months [9]. It was later reported that there was no significant difference in hernia recurrence after five years between the two groups [10]. In this case, SIS was chosen because it is absorbable; therefore the reinforcement would be present for the crucial period immediately after surgery, but absorbed later to prevent other mesh-related complications. Because short-term benefits derived from reinforcement with SIS would be erased after five years, a non-absorbable biological mesh should provide better long-term results.

In this case I selected a non-cross-linked acellular tissue matrix derived from porcine dermis that supports tissue regeneration and undergoes rapid revascularization without a significant inflammatory or immunological response in animal models [5,12,13]. This should lower the rate of long-term complications such as infection and erosion. Non-cross-linked PADMs have been shown to cause fewer adhesions than synthetic meshes [14] or cross-linked PADMs [15]. Our previous experience with Strattice™ grafts in abdominal wall repair led us to utilize this graft to reinforce the primary hiatal hernia repair. Despite the thinness of the crura, a primary repair of the hiatal hernia was initially attempted. It is important to always attempt a primary repair when employing biological grafts in order to provide the graft with a matrix to begin the regeneration process.

The most commonly used graft shapes tend to be a U shape which partially surrounds the esophagus, a rectangular mesh fixed to both the right and left crus, and the keyhole that completely surrounds the esophagus. I consider the placement of the mesh in a U shape to be extremely important because circumferential graft placement can lead to swallowing issues if the graft contracts. It is important to achieve a good overlay of the graft without using excess material. The suture line from the primary hiatal closure can cause a ridge that makes sufficient overlay of the graft more difficult. Sutures were used to initially secure the graft; however, optimal tissue apposition was achieved with fibrin glue. This prevents migration or slippage of the graft in the dynamic environment of the hiatus.

Complications arising from the Nissen fundoplication required the patient to undergo a second surgery to repair the slipped wrap. It is difficult to determine the exact cause of the slipped wrap; however, it is possibly related to the construction of the original wrap. The follow-up surgery allowed for observation of the progress of the hiatal repair 11 months after the initial surgery. There were a few adhesions that were taken down with a suction device. The graft was well incorporated overlying the hiatus and actually looked like normal fascia with no sign of graft contraction. The hiatus did dilate anterior to the repair; however, the portion reinforced with the non-cross-linked PADM was still intact. Dilation anterior to the graft is a common issue in these cases: placing the graft in a C shape might provide better reinforcement of the hiatus and prevent this complication. Strattice™ is non-absorbable and quickly vascularizes, making it ideal for reinforcement of a hiatal hernia repair when dealing with the dynamic environment around the esophagus.

## Conclusion

Acellular tissue matrices have been shown to be safe and effective for abdominal wall repair. Although there is a paucity of data on the long-term outcomes of biological grafts at the esophageal hiatus they have been successful at lowering the rate of recurrence in the short term. This case report shows that in this case the non-cross-linked PADM did reinforce the hiatal hernia repair without additional mesh-related complications. Strattice™ was chosen because it is non-absorbable and should provide long-term reinforcement of the paraesophageal hernia repair. The graft was intact and well incorporated overlying the hiatal repair 11 months after the initial surgery. Proper technique when placing the graft is important: good overlay, efficient tissue apposition, and placing the graft in a non-circumferential manner are essential to a successful reinforcement of the primary hiatal repair. More studies are needed to support the claim that there are long-term benefits to this approach to hiatal hernia repair and use of non-cross-linked PADMs in this application.

## Consent

Written informed consent was obtained from the patient for publication of this case report and accompanying images. A copy of the written consent is available for review by the Editor-in-Chief of this journal.

## Competing interests

The author declares that there are no competing interests.
